# LRP1 Activation Promotes Metabolic Reprogramming and Mrc1 Expression to Attenuate LPS‐Induced Cognitive Deficits: An Integrated Omics Analysis

**DOI:** 10.1002/cns.70950

**Published:** 2026-05-29

**Authors:** Mengyao Qu, Yanan He, Lu Yu, Huikai Yang, Yixun Lu, Yingfu Li, Rui Wang, Miao Sun, Yulong Ma, Weidong Mi

**Affiliations:** ^1^ Department of Anesthesiology, the First Medical Center Chinese PLA General Hospital Beijing China; ^2^ Medical School of Chinese PLA General Hospital Beijing China

**Keywords:** cognitive impairment, insulin resistance, LRP1, multi‐omics

## Abstract

**Aim:**

Central insulin resistance and neuroinflammation act as synergistic drivers in the pathogenesis of cognitive decline. While the low‐density lipoprotein receptor‐related protein 1 (LRP1) is known to maintain blood–brain barrier integrity, its capacity to decouple the inflammation‐metabolism axis remains underexplored. This study investigates whether activation of LRP1 can ameliorate LPS‐induced cognitive deficits by recalibrating cerebral glucose metabolism.

**Methods:**

We utilized an integrative approach combining behavioral phenotyping with targeted metabolomics and transcriptomics to dissect the neuroprotective mechanism of SP16, a selective LRP1 agonist. Cognitive dysfunction was modeled in mice via intracerebroventricular (i.c.v) LPS administration, followed by systemic intervention with intraperitoneally (i.p) injected SP16.

**Results:**

SP16 treatment preserved cognitive function and prevented neuronal structural atrophy against the LPS injection. Mechanistically, LRP1 activation did more than suppress inflammation; it functionally modulated hippocampal insulin sensitivity and re‐established redox homeostasis. Crucially, metabolomic profiling highlighted a restoration of glycolytic flux, centered on the normalization of fructose‐1,6‐bisphosphate (FBP) levels. This metabolic reprogramming coincided with the upregulation of the M2‐like reparative marker, Mrc1.

**Conclusion:**

Our findings identify LRP1 as a regulator that bridges metabolic health and immune resolution. By enforcing a metabolic shift via the FBP node, SP16 effectively guides microglia from a pro‐inflammatory state toward tissue repair. Thus, honing in on SP16‐mediated metabolic reprogramming presents an opportunity for a therapeutic intervention against neuroinflammation‐associated cognitive impairment, offering a more nuanced alternative to broad‐spectrum anti‐inflammatories.

## Graphical abstract

LPS triggers neuroinflammation within the central nervous system. Conversely, the activation of LRP1 by SP16 mitigates insulin resistance, improves glucose metabolism, and facilitates the polarization of microglia toward an anti‐inflammatory M2‐like phenotype, which collectively contribute to the restoration of cognitive function.

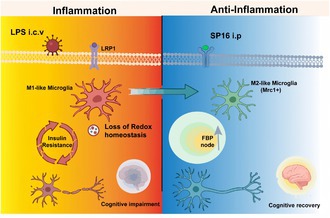

## 1 Introduction

Cognitive impairment, a symptom that gradually worsens with neurodegenerative conditions like age‐related cognitive decline, Alzheimer's disease, and perioperative neurocognitive disorders, places a significant strain on society [1, 2]. The treatment options for these conditions are still scarce, and a comprehensive comprehension of their fundamental mechanisms is still absent. It should be noted that neuroinflammation, characterized by the activation of glial cells and the release of inflammatory factors within the central nervous system (CNS), is increasingly acknowledged as a central factor in the development and advancement of cognitive disorders [3, 4, 5]. Neuroinflammatory mechanisms could potentially contribute to cognitive decline via a network of interconnected pathways, such as synaptic malfunction, disrupted neurotransmission, mitochondrial dysfunction, and oxidative stress [6, 7, 8, 9]. Considering that neuroinflammation is a possible treatment target for cognitive decline, a variety of interventions for postoperative neurocognitive dysfunction (PND) have been studied in both animal models and clinical trials [10, 11]. However, the underlying pathological mechanisms by which neuroinflammation contributes to PND remain incompletely understood. Additional investigation focusing on inflammation‐induced neuronal dysfunction is crucial in mitigating the public health and economic impact of PND.

Lipopolysaccharide (LPS) is an endotoxin derived from the outer membrane of gram‐negative bacteria, which functions as a ligand for toll‐like receptor 4 (TLR4). Upon binding to LPS, TLR4 triggers signaling cascades from the plasma membrane, followed by internalization into endosomal compartments [12]. LPS has been extensively used to induce animal models of peripheral and central inflammation, largely due to its ability to trigger the nuclear factor kappa B (NF‐κB) pathway, thereby stimulating the production and release of cytokines and reactive oxygen species (ROS) [13, 14, 15]. In mice, cognitive deficits can be induced by LPS through several routes of administration, such as intraperitoneal (i.p.) or intracerebroventricular (i.c.v.) injection [15, 16]. In our previous study, we found LPS i.c.v. Injections in mice induced cognitive impairment and insulin resistance (IR) [17]. Activating the insulin signaling pathway can effectively improve cognitive deficits and reduce neuroinflammation [18]. Consequently, IR represents a potential therapeutic target for ameliorating neuroinflammation‐induced cognitive decline.

Low‐density lipoprotein receptor‐related protein 1 (LRP1) is a ubiquitously expressed receptor within the low‐density lipoprotein receptor family, present on the surface of diverse cell types such as hepatocytes, macrophages, neurons, and glial cells [19]. A considerable amount of evidence indicates that membrane‐bound LRP1 exerts a robust anti‐inflammatory effect [20, 21, 22]. By interacting with its co‐receptor systems, LRP1 counteracts inflammatory cytokines and toll‐like receptors, underscoring its pivotal role in innate immune regulation [23]. Conditional deletion of LRP1 in neurons or glial cells induces neuroinflammation. Notably, neuronal LRP1 deficiency aggravates insulin resistance, disrupts glucose metabolism, and contributes to synaptic dysfunction, changes that may represent early pathological alterations occurring prior to or during the onset of cognitive decline [24]. In microglia, LRP1 has been implicated in the progression of experimental autoimmune encephalomyelitis [25]. It also modulates c‐Jun N‐terminal kinase (JNK) and NF‐κB signaling pathways to suppress microglial activation [26]. Based on these findings, we propose that pharmacological activation of LRP1 may alleviate cognitive dysfunction induced by LPS.

Given the established roles of LRP1 in modulating inflammation and insulin resistance, we hypothesized that pharmacological activation of LRP1 might attenuate LPS‐induced neuroinflammation and associated cognitive deficits. In this study, we evaluated the effects of SP16, a selective LRP1 agonist, in an LPS challenge model. By clarifying the neuroprotective mechanisms of LRP1, this work seeks to propose a novel therapeutic approach for mitigating brain insulin resistance and preventing clinically significant cognitive decline.

## 2 Materials and Methods

### 2.1 Animal Treatments and Experimental Design

Male C57BL/6J mice, aged 8–10 weeks (body weight 20–25 g), were group‐housed (4–5 per cage) under standard conditions with a constant temperature of 22°C ± 3°C, humidity of 50%–70%, and a 12‐h light/dark cycle. After one week of acclimatization with free access to food and water, a cognitive impairment model was established by intracerebroventricular single injection of lipopolysaccharide (LPS), in accordance with established protocols [18]. LPS (Sigma, USA) was dissolved in artificial cerebrospinal fluid (Biofount, China) to obtain a 1 mg/mL solution. SP16 peptide (12 μg) was administered intraperitoneally at three time points relative to the LPS challenge: 2 h pre‐injection (−2 h), immediately prior (0 h), and 30 min post‐injection (+0.5 h) [27]. At exactly 24 h post‐injection, the cognitive functions of the mice were evaluated using the Morris water maze and the Y‐maze test.

The peptide SP16 (Ac‐VKFNKPFVFLNleIEQNTK‐NH_2_) was purchased from Peptidesbank (Hefei, Anhui, China). It was synthesized and confirmed to have a purity exceeding 95% through analysis by high‐performance liquid chromatography (HPLC) and mass spectrometry (MS).

### 2.2 Morris Water Maze (MWM)

The Morris water maze (MWM) test was performed as previously described [28]. Briefly, mice underwent a 5‐day acquisition training protocol, performing four trials each day. Intracerebroventricular injection was conducted on day 6. Twenty‐four hours after surgery, a probe test was administered, during which the platform was removed, and mice were allowed 60 s to freely explore the pool. The following parameters were recorded: The average escape latency across the training phase, as well as the time spent in the target quadrant and the swimming distance during the probe test. All data acquisition and analysis were performed by researchers blinded to the group assignments.

### 2.3 Y Maze

The Y‐maze test was employed to evaluate hippocampal‐dependent spontaneous spatial recognition [29]. The maze consisted of three identical arms (each 45 cm long) oriented at 120° angles. Distinct visual cues were positioned at the distal end of each arm. Following a protocol adapted from prior work, a 5‐min acquisition trial was conducted 24 h after surgery, in which one arm (designated as the “closed/novel” arm) was blocked. After a 2‐h interval, mice were returned to the maze for a 5‐min test trial with all arms open. We recorded the time spent in the newly accessible (novel) arm and the number of entries into it (an entry was defined as all four limbs crossing the arm threshold). The assignment of old and novel arms was randomized using Excel, and all data were collected and analyzed by investigators blinded to group assignment.

### 2.4 Hematoxylin–Eosin (H&E) Staining and Nissl Staining

To assess hippocampal pathology and neuronal morphology, we performed hematoxylin and eosin (H&E) and Nissl staining 24 h after LPS injection (*n* = 3), following established procedures. Briefly, mice were anesthetized via intraperitoneal injection of 1% pentobarbital sodium (40 mg/kg) and transcardially perfused with ice‐cold heparinized saline, followed by 4% paraformaldehyde. The brains were paraffin‐embedded, and coronal sections (4 μm thick) containing the hippocampal region were prepared. Staining was carried out using commercial H&E and Nissl staining kits (Wuhan Servicebio Technology Co. Ltd., China) according to the manufacturer's instructions. Quantification of HE‐ and Nissl‐positive areas in the hippocampus was performed by investigators blinded to group assignment using a light microscope (BX51; Olympus, Tokyo, Japan).

### 2.5 Immunofluorescence Staining

Immunofluorescence staining was performed according to standard protocols. Brains were collected 24 h after surgery, following transcardial perfusion with ice‐cold saline and fixation with 4% paraformaldehyde. Coronal sections (10 μm thick) containing the hippocampus (*n* = 3) were prepared using a Leica CM1900 cryostat. Sections were incubated with primary antibodies against GFAP (1:200, Cell Signaling Technology, USA), Iba1 (1:200, Cell Signaling Technology, USA), and Mrc1 (1:200, abcam, USA), followed by appropriate Alexa‐594‐conjugated and Alexa‐488‐conjugated secondary antibodies (1:800, donkey anti‐mouse, Invitrogen). Images were acquired using a fluorescence microscope (RON‐K; Echo, USA) and analyzed by an observer blinded to group allocation.

TUNEL (Terminal deoxynucleotidyl transferase‐mediated dUTP nick end labeling) staining was performed using an in situ cell death detection kit (e.g., Roche, Basel, Switzerland) according to the manufacturer's instructions. Briefly, paraffin‐embedded hippocampal sections were deparaffinized in xylene and rehydrated through a graded ethanol series. The sections were then incubated with proteinase K (20 μg/mL) for 15–20 min at 37°C to permeabilize the tissue. After washing with phosphate‐buffered saline (PBS), endogenous peroxidase activity was blocked with 3% H₂O₂ in methanol for 10 min at room temperature. The sections were subsequently incubated with the TUNEL reaction mixture containing terminal deoxynucleotidyl transferase (TdT) and fluorescein‐labeled dUTP for 60 min at 37°C in a humidified dark chamber. Negative controls were prepared by omitting the TdT enzyme from the reaction mixture. After washing with PBS, the sections were incubated with converter‐peroxidase (anti‐fluorescein antibody conjugated with horseradish peroxidase) for 30 min at 37°C. The signal was visualized using 3,3′‐diaminobenzidine (DAB) as the chromogen, resulting in brown nuclear staining of apoptotic cells. Finally, sections were counterstained with hematoxylin, dehydrated, and mounted for light microscopy observation.

Reactive Oxygen Species (ROS) Measurement: Intracellular ROS levels were assessed using Dihydroethidium (DHE) staining. Briefly, hippocampal sections were incubated with 10 μM DHE (Thermo Fisher Scientific, USA) for 30 min at 37°C in the dark. Fluorescence was observed under a fluorescence microscope (BX51; Olympus, Tokyo, Japan). Images were captured, and fluorescence intensity was quantified using ImageJ software.

### 2.6 Western Blot Analysis

At the designated time points, mice were humanely euthanized under general anesthesia via cervical dislocation and immediately perfused transcardially with ice‐cold phosphate‐buffered saline (PBS). Brains were rapidly removed, rinsed briefly in chilled saline, and placed on ice. Hippocampi were then carefully dissected bilaterally, snap‐frozen in liquid nitrogen, and stored until use. For each sample, both hippocampi from the same animal were pooled, lysed, and subjected to western blot analysis as described previously. The following primary antibodies were used: LRP1 (1:1000, Cell Signaling Technology, USA), GFAP (1:1000, Cell Signaling Technology, USA), Iba1 (1:1000, Cell Signaling Technology, USA), anti‐p‐PI3K (1:1000, Abcam, USA), anti‐PI3K (1:1000, Abcam, USA), anti‐p‐AKT (1:1000, Abcam, USA), anti‐AKT (1:1000, Abcam, USA), anti‐p‐GSK‐3β (1:1000, Abcam, USA), anti‐GSK‐3β (1:1000, Abcam, USA), anti‐β‐actin (1:8000, Abcam, USA), and Mrc1 (1:1000, Abcam, USA). Protein bands were visualized using the LI‐COR Odyssey System (LI‐COR Biotechnology, USA), and band intensities were quantified with NIH ImageJ software. All densitometric analyses were performed by investigators blinded to group assignment.

### 2.7 Detection of ELISA Levels

Enzyme‐linked immunosorbent assay (ELISA) kits (Jiangsu Meimian Biotechnology Co. Ltd., Jiangsu, China) were used to detect the secretion of pro‐inflammatory cytokines (interleukin‐6 (IL‐6), interleukin‐1β (IL‐1β), and tumor necrosis factor‐α (TNF‐α)), oxidative stress substance (H_2_O_2_), and antioxidant‐related indicators, including superoxide dismutase (SOD), glutathione (GSH), and total antioxidant capacity (T‐AOC). The absorbance was measured at 450 nm using a full‐wavelength microplate reader (Thermo, USA), and the absorbance of each sample was compared to a standard curve to calculate the concentrations of the measured factors.

### 2.8 RT‐qPCR

Total RNA was extracted from brain tissue using TRIzol reagent (Invitrogen, St. Louis, MO, USA) following the manufacturer's protocol. For each sample, 1 μg of RNA was reverse‐transcribed to cDNA using the PrimeScript RT reagent kit (Vazyme Bio Inc., Nanjing, China). Quantitative reverse transcription‐polymerase chain reaction (RT‐qPCR) was then performed with PowerUp SYBR Green Master Mix (Thermo Fisher Scientific Inc., U.S.A) on a 7500 Real‐Time PCR System (Applied Biosystems). Reactions were carried out in a final volume of 10 μL containing 5 μL of PCR mix, 1 pmol each of forward and reverse primers, 1 μL of cDNA template, and nuclease‐free water. The thermal cycling protocol consisted of an initial denaturation at 95°C for 20 s, followed by 40 cycles of 95°C for 20 s, 60°C for 30 s, and 72°C for 25 s. All reactions were run in triplicate. Threshold cycle (CT) values were recorded, and the relative mRNA expression of target genes was calculated using the 2‐^ΔΔCT^ method, with β‐actin serving as the internal control. mRNA levels are presented as fold changes relative to the sham group. The sequences of the primer pairs for target genes are shown below:

**Table:** 

Gene	Forward primer sequence (5′‐3′)	Reverse primer sequence (5′‐3′)
*β‐Actin*	GTGACGTTGACATCCGTAAAGA	GCCGGACTCATCGTACTCC
*IL‐6*	TAGTCCTTCCTACCCCAATTTCC	TTGGTCCTTAGCCACTCCTTC
*IL‐1β*	CAGGATGAGGACATGAGCACC	CTCTGCAGACTCAAACTCCAC
*TNF‐α*	CCCTCACACTCAGATCATCTTCT	GCTACGACGTGGGCTACAG
*Mrc1*	CTCTGTTCAGCTATTGGACGC	TGGCACTCCCAAACATAATTTG
*Slc16a4*	TGGAATGGGGCTGACATTTTT	ATTGGCTGTGGATGGGTCTTA
*P2ry13*	CTCTGGGTGTTCGTCCACATC	GTGTGAGTCGGAAAGGATTTTG
*Clec7a*	AAAGCCAAACATCGTCTCACC	CGAGTTGGGGAAGAATGCTGA
*Cxcr4*	GACTGGCATAGTCGGCAATG	AGAAGGGGAGTGTGATGACAA

### 2.9 Targeted Metabolomics Analysis

Targeted metabolomic analysis of glucose metabolism was performed by OBio Technology Co. Ltd. (Shanghai, China). Briefly, hippocampal tissues harvested 24 h post‐injection (*n* = 5) were weighed (50 mg/sample) and subjected to metabolite extraction for LC–MS analysis. The downstream data processing pipeline encompassed quality control validation, multivariate statistical analysis, differential metabolite screening, and metabolic pathway enrichment.

### 2.10 Transcriptomics

Transcriptomic analysis was conducted on brain tissue samples (100–200 mg) from the same anatomical region of mice per group. Total RNA was isolated via the Trizol method and assessed using a NanoDrop ND 2000. Only high‐quality RNA (OD260/280 = 1.8–2.2, OD260/230 ≥ 2.0, RIN ≥ 6.5, 28S:18S ≥ 1.0, > 1 μg) was utilized. Sequencing libraries were constructed from 1 μg RNA using the Illumina TruSeq RNA Sample Preparation Kit. Roughly 300 bp cDNA fragments were selected on a 2% agarose gel and amplified via 15 PCR cycles using Phusion DNA polymerase. Libraries were quantified with a TBS380 fluorometer and paired‐end sequenced (2 × 150 bp) on an Illumina HiSeq xten/NovaSeq 6000 platform.

Gene expression was quantified as FPKM on the Majorbio Cloud Platform. Following quality control, differentially expressed genes (DEGs) were identified using DESeq2 (*p* < 0.05, fold change ≥ 2). Significant DEGs underwent GO and KEGG functional enrichment. Pearson correlation evaluated associations between gene expression and metabolite levels, with *p* < 0.05 indicating statistical significance.

### 2.11 Statistical Analysis

All continuous data are presented as mean ± standard error of the mean (SEM). The assumption of normality was evaluated using the Shapiro–Wilk test, and homogeneity of variances was verified using Levene's test. For data meeting both assumptions, comparisons between two groups were performed using an unpaired, two‐sided Student's *t*‐test, while comparisons among three or more groups were analyzed using one‐way analysis of variance (ANOVA) followed by Tukey's post hoc test. If the data failed the normality test, the non‐parametric Mann–Whitney U test was applied. If equality of variances was violated, Welch's *t*‐test or Welch's ANOVA was used. All statistical tests were two‐sided, and a *p*‐value < 0.05 was considered statistically significant. Graphs were generated using GraphPad Prism 9.0.

## 3 Results

### 3.1 SP16 Significantly Improved LPS‐Induced Cognitive Impairment

**FIGURE 1:**
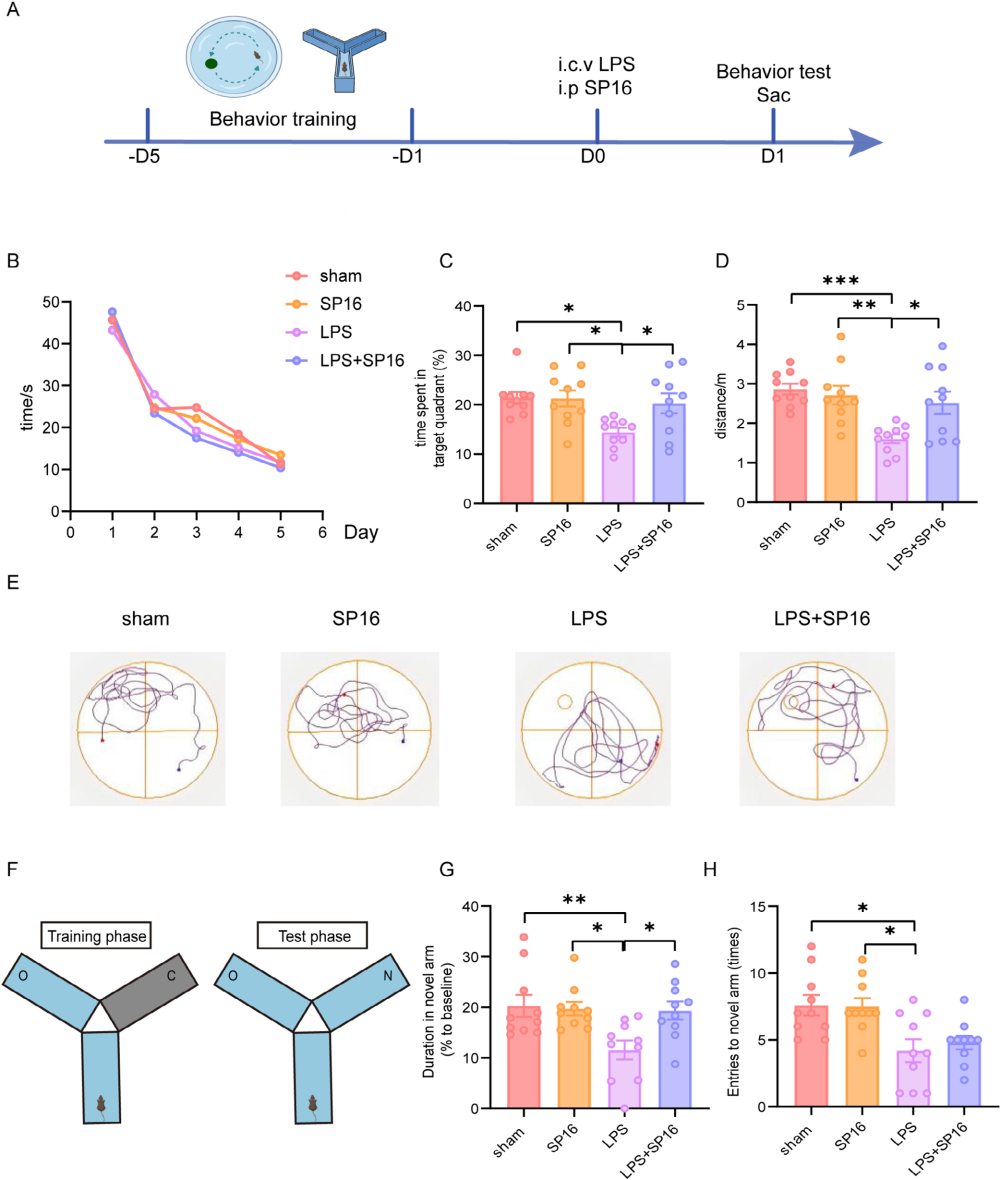
SP16 ameliorates LPS‐induced cognitive impairment. (A) Schematic timeline of the experimental design and drug administration. (B–E) Evaluation of spatial memory using the Morris Water Maze (MWM) (*n* = 10). Experts examined (B) escape latency during the 5‐day training phase, (C) percentage of time spent in the target quadrant during the probe trial, (D) total swimming distance, and € representative swimming trajectories from each group. (F–H) Assessment of working memory using the Y‐maze (*n* = 10). (F) Schematic illustration of the Y‐maze task. (G) Number of entries into the novel arm. (H) Distance traveled within the novel arm. O: Old arm; C: Closed arm; N: Novel arm. Data are presented as mean ± SEM. *p < 0.05, **p < 0.01, ***p < 0.001.

To investigate the effects of SP16, we evaluated cognitive function using the MWM and Y‐maze tests. Figure 1A shows the behavioral test design and treatment schedule. During the training period, mice across experimental groups exhibited comparable escape latency times (Figure 1B). The movement of all mice was analyzed statistically, and the traveling trajectory in the LPS group was mixed (Figure 1E), suggesting LPS treatment decreased spatial memory ability. The probe test revealed significant cognitive impairment in LPS‐treated animals compared to the sham group, evidenced by reduced time spent in the target quadrant (*p <* 0.05) (Figure 1C) and the total traveled distance (*p <* 0.001) (Figure 1D). In contrast, the LPS + SP16 group demonstrated increased traveled duration (*p <* 0.05) and total traveled distance (*p <* 0.05) compared with the LPS group (Figure 1C,D). No changes were observed between the sham group and the SP16 group, suggesting that SP16 can improve cognition to some extent. Compared with the sham group, the speed of the LPS group was lower (*p* < 0.01) (Figure S1A). Meanwhile, the platform crossing times in the LPS group were significantly lower than those in the sham (*p* < 0.01), SP16 (*p* < 0.01), and LPS + SP16 (*p* < 0.05) groups, suggesting impaired spatial memory (Figure S1B).

In the Y‐maze test (Figure 1F), the LPS group showed a significantly decreased percentage of entries to the novel arm (*p <* 0.05) and duration in the novel (*p <* 0.01) arm compared with the sham group (Figure 1G,H). Conversely, the LPS + SP16 group exhibited a significantly elevated percentage duration in the novel arm compared with the LPS group (*p <* 0.05). However, significant differences were not observed in the entries to the novel arm between the LPS group and the LPS + SP16 group. The total entries of LPS and LPS + SP16 were lower than those in sham and SP16 (both *p* < 0.01), indicating severe acute sickness behavior and suppressed exploratory drive (Figure S1C).

Taken together, these behavioral data demonstrate that SP16 selectively attenuates LPS‐induced spatial memory impairment, even though the generalized acute sickness behavior remains partially present at 24 h post‐insult.

### 3.2 SP16 Treatment Dramatically Attenuated LPS‐Induced Neural Damage in the Hippocampus

**FIGURE 2:**
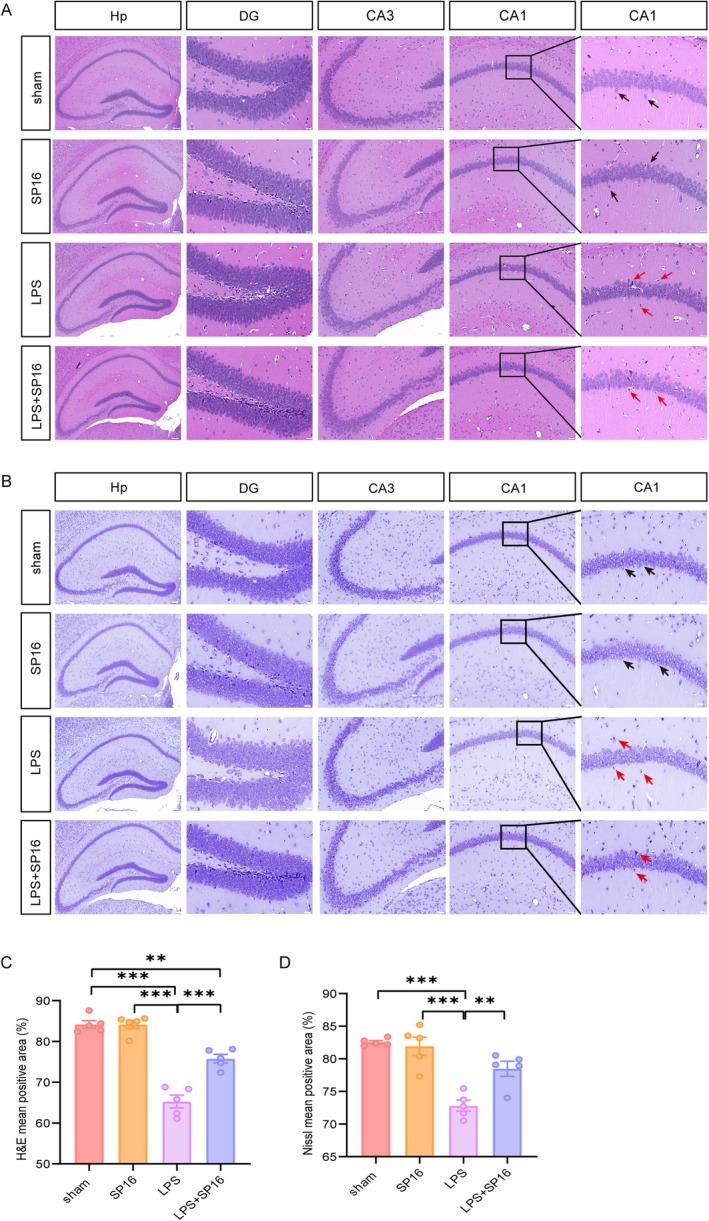
SP16 attenuates LPS‐induced neuronal damage in the hippocampus. Histological assessment (H&E and Nissl staining) was performed in the DG, CA3, and CA1 regions (*n* = 3). (A) Representative H&E staining showing cell morphology 24 h post‐LPS injection. Black arrows indicate healthy cells with compact structure and clear outlines; red arrows indicate damaged cells characterized by sparse arrangement, fuzzy outlines, and structural disarray. (B) Nissl staining revealing neuronal viability. Black arrows point to intact neurons with plump cell bodies; red arrows highlight injured neurons exhibiting cell body shrinkage and nuclear pyknosis. (C, D) Quantitative analysis of the mean positive area for (C) H&E and (D) Nissl staining across the four groups. Data are presented as mean ± SEM. **p < 0.01, ***p < 0.001. Abbreviations: HE, hematoxylin–eosin; DG, dentate gyrus; CA, cornu ammonis.

To assess the neuronal damage and pathology in the hippocampus after LPS administration, H&E and Nissl staining were conducted. As shown in Figure 2A, normal cells in H&E staining showed complete borders and structures with clear nucleoli, while damaged cells lost cytoplasm and had irregular, condensed nucleoli. Histological analyses using H&E and Nissl staining demonstrated severe LPS‐induced neuronal injury, which was effectively mitigated by SP16 treatment. Morphologically, normal neurons exhibited intact, plump cell bodies, whereas injured neurons in the LPS group displayed shrunken, pyknotic nuclei and fragmented soma (Figure 2B). Quantitatively, LPS exposure profoundly reduced the mean area of both H&E‐positive cells (*p* < 0.001 vs. sham and SP16 groups) and Nissl‐positive cells (*p* < 0.001 vs. sham and SP16 groups). Co‐treatment with SP16 significantly attenuated these reductions, increasing the viable cell area in both H&E (*p* < 0.01) and Nissl (*p* < 0.01) evaluations compared to the LPS‐alone group (Figure 2C,D). Notably, although SP16 markedly improved neuronal survival, the H&E‐positive area in the LPS + SP16 group remained slightly lower than that of the sham control (*p* < 0.01).

To further evaluate neuronal apoptosis in the hippocampus, TUNEL staining was performed. Figure S1D shows the proportion of TUNEL‐positive cells in the sham, SP16, LPS, and LPS + SP16 groups. Compared with the sham group, intracerebroventricular injection of LPS significantly increased the number of TUNEL‐positive cells (*p* < 0.001); after SP16 intervention, the number of positive cells was markedly reduced compared with the LPS group (*p* < 0.05), indicating that neuronal apoptosis was significantly inhibited (Figure S1E).

### 3.3 SP16 Markedly Inhibited LPS‐Induced Neuroinflammation

**FIGURE 3:**
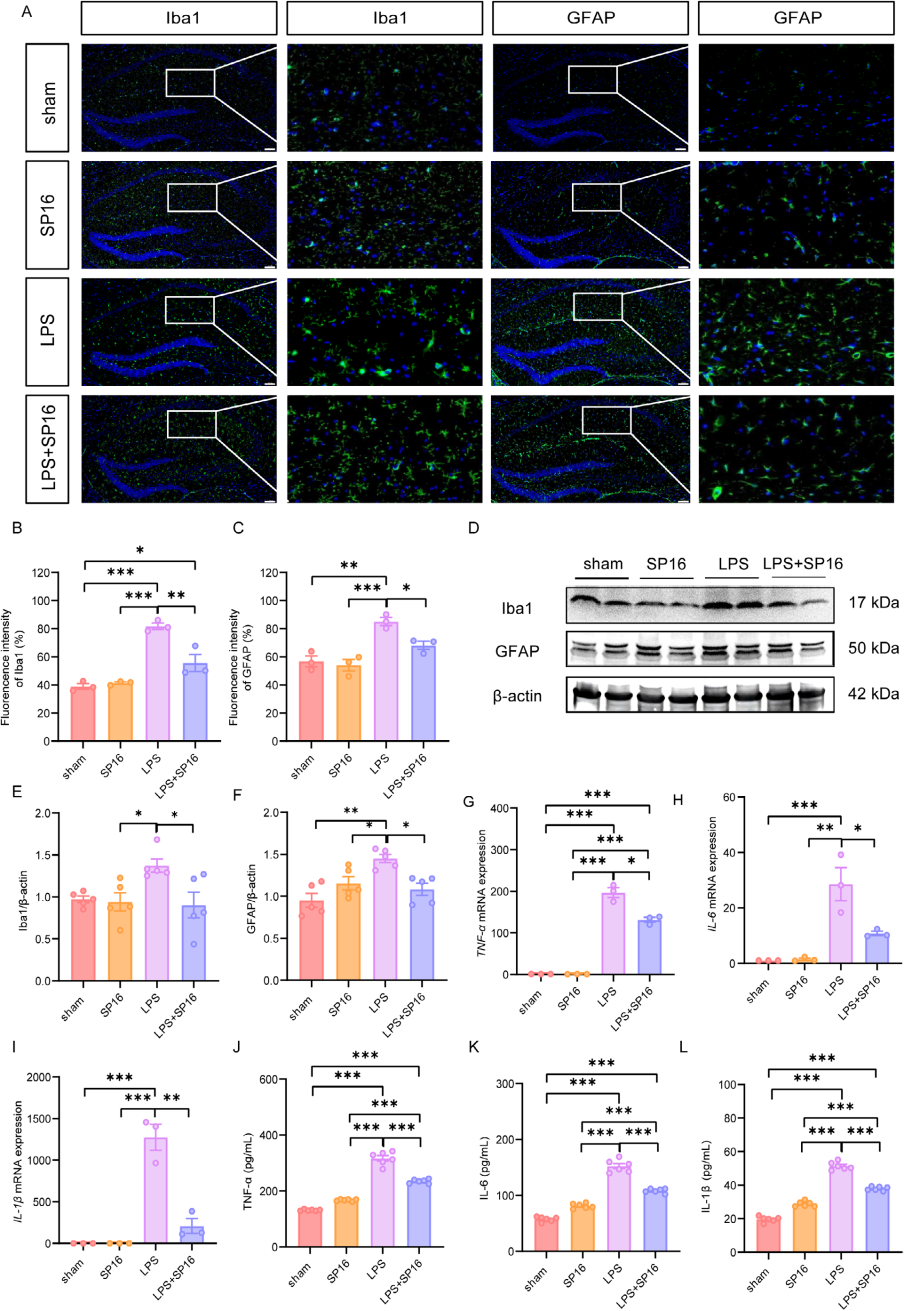
SP16 mitigates LPS‐induced microglial and astrocytic activation in the hippocampus. All analyses were performed on hippocampal tissues collected 24 h post‐LPS injection. (A) Representative immunofluorescence staining for microglia (Iba1) and astrocytes (GFAP). Scale bars = 50 μm. (B, C) Quantification of fluorescence intensity indicating the activation state of microglia and astrocytes. (D) Representative Western blot bands for Iba1 and GFAP protein expression. (E, F) Densitometric quantification of Iba1 and GFAP protein levels relative to control. (G–I) Relative mRNA expression levels of pro‐inflammatory cytokines (*TNF‐α, IL‐6*, and *IL‐1β*). (J–L) Protein concentrations of TNF‐α, IL‐6, and IL‐1β were determined by ELISA. Data are presented as mean ± SEM (*n* = 3). *p < 0.05, **p < 0.01, ***p < 0.001. Iba1, ionized calcium‐binding adapter molecule 1; GFAP, glial fibrillary acidic protein.

To characterize the neuroinflammatory response, we first examined glial reactivity in the hippocampus. Representative immunofluorescence images (Figure 3A) revealed that LPS injection triggered robust activation of both microglia and astrocytes compared to the sham (Iba1: *p* < 0.001; GFAP: *p* < 0.01) and SP16 groups (both *p* < 0.001) (Figure 3B,C). Conversely, co‐treatment with SP16 significantly reversed this trend, suppressing the abnormal activation of both Iba1 (*p* < 0.01) and GFAP (*p* < 0.05). This morphological evidence was corroborated by Western blot analysis (Figure 3D–F). Consistently, LPS exposure drastically elevated the protein expression of Iba1 (both *p* < 0.05 vs. sham and SP16 groups) and GFAP (*p* < 0.01 vs. sham and *p* < 0.05 SP16 groups). Crucially, SP16 administration effectively mitigated this gliosis, reducing the protein levels of both activation markers back toward baseline (*p* < 0.05 vs. LPS group).

We next profiled the inflammatory cytokine milieu driven by this glial activation. Parallel analyses using RT‐qPCR and ELISA demonstrated that LPS challenge induced a “cytokine storm”, characterized by a surge in TNF‐α, IL‐1β, and IL‐6 at both mRNA (Figure 3G–I) and protein levels (Figure 3J–L). At the mRNA level, LPS challenge significantly upregulated the expression of pro‐inflammatory cytokines (all *p* < 0.001 vs. sham and SP16 groups). Similarly, protein levels of these mediators were drastically elevated following LPS exposure (all *p* < 0.001). Conversely, SP16 treatment profoundly dampened this inflammatory cascade, significantly downregulating both the transcription and secretion of these key cytokines compared to the LPS group (mRNA: *p* < 0.05; protein: *p* < 0.001). Notably, while SP16 markedly attenuated the neuroinflammatory response, the levels of these cytokines in the LPS + SP16 group remained significantly higher than those in the sham and SP16 groups (all *p* < 0.001), indicating a partial but robust rescue effect. Collectively, these data indicate that SP16 exerts potent anti‐inflammatory effects by suppressing glial activation and downstream cytokine production.

### 3.4 SP16 May Alleviate Neuroinflammation via the PI3K/AKT/GSK‐3β Pathway

**FIGURE 4:**
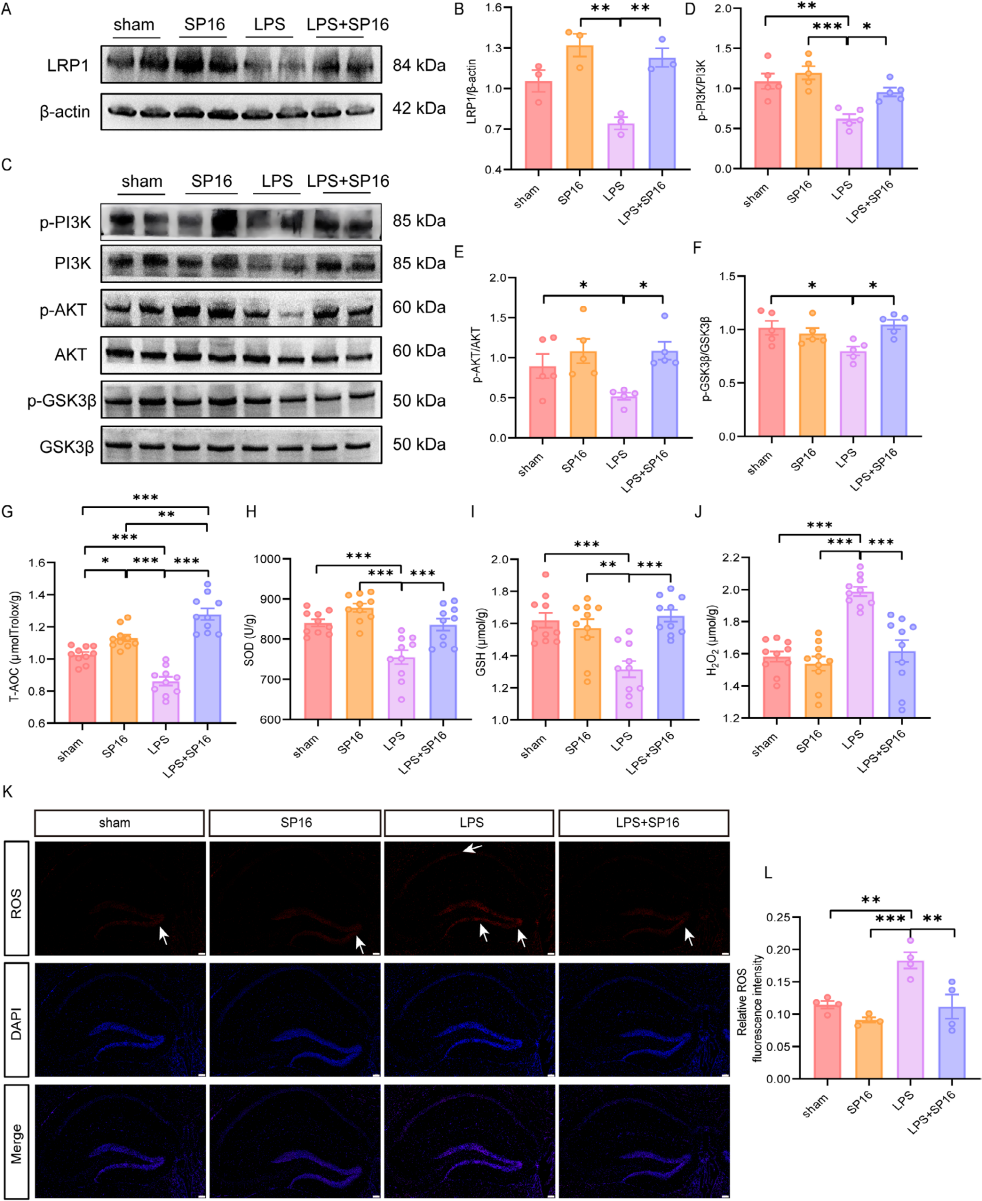
SP16 may alleviate neuroinflammation via the PI3K/AKT/GSK‐3β pathway. Measurements were performed on hippocampal tissues 24 h post‐LPS injection. (A, B) Representative Western blots and quantitative analysis of LRP1 expression (*n* = 3). (C) Representative Western blot bands for the downstream PI3K/AKT/GSK‐3β pathway. (D–F) Densitometric quantification of the phosphorylation ratios for (C) p‐PI3K/PI3K, (E) p‐AKT/AKT, and (F) p‐GSK‐3β/GSK‐3β. (G–J) Evaluation of oxidative stress markers. Levels of T‐AOC, SOD, GSH, and H_2_O_2_ in brain tissue were measured by ELISA. (K, L) Visualization of oxidative stress. (K) Representative immunofluorescence images of ROS staining and (L) corresponding quantification of fluorescence intensity. Scale bars = 50 μm. Data are presented as mean ± SEM. *p < 0.05, **p < 0.01, ***p < 0.001.

Mechanistically, we first evaluated the LRP1‐associated signaling axis. Western blot analysis revealed that while SP16 administration alone upregulated LRP1, LPS challenge led to a marked attenuation of its expression (Figure 4A). In the combined treatment group, SP16 exhibited a tendency to restore LRP1 levels against LPS‐induced suppression, and this trend reached statistical significance (Figure 4B).

We further interrogated the downstream insulin signaling pathway (PI3K/AKT/GSK‐3β). As shown in (Figure 4C–F), LPS exposure significantly suppressed this vital signaling axis, evidenced by markedly reduced phosphorylation ratios of p‐PI3K/PI3K (*p* < 0.01 vs. sham; *p* < 0.001 vs. SP16), p‐AKT/AKT (*p* < 0.05 vs. sham), and p‐GSK‐3β/GSK‐3β (*p* < 0.05 vs. sham). Importantly, co‐administration of SP16 effectively reversed these LPS‐induced deficits, significantly restoring the phosphorylation levels across all three targets (all *p* < 0.05 compared to the LPS group).

We next evaluated the impact of this signaling modulation on oxidative status. Biochemical assays demonstrated that LPS treatment severely compromised antioxidant defenses, leading to reductions in T‐AOC, SOD, and GSH, alongside elevated H_2_O_2_ levels (Figure 4G–J). Notably, SP16 administration effectively ameliorated these biochemical deficits. LPS exposure induced oxidative damage, evidenced by a dramatic increase in H_2_O_2_ levels accompanied by significant depletions in antioxidant reserves, including SOD and GSH (all *p* < 0.001 vs. sham). Treatment with SP16 effectively reversed these pathological trends, significantly suppressing H_2_O_2_ accumulation and restoring SOD and GSH levels. Notably, a distinct regulatory pattern was observed for T‐AOC (Figure 4G). While LPS drastically reduced T‐AOC (*p* < 0.001 vs. sham), co‐treatment with SP16 rescued this decline (*p* < 0.001 vs. LPS). Interestingly, SP16 administration alone induced a mild basal increase in T‐AOC compared to the sham group (*p* < 0.05), suggesting that while SP16 generally maintains physiological homeostasis without causing toxicity, it may proactively prime baseline antioxidant defenses. Furthermore, although the LPS + SP16 group exhibited significantly elevated T‐AOC, statistical differences remained when compared to the SP16 group (*p* < 0.01). Taken together, these data indicate that while SP16 effectively modulates the PI3K/AKT pathway to counteract neuroinflammation and oxidative stress, it acts as a potent pharmacological buffer rather than fully neutralizing the severe LPS stimulus.

We also performed DHE staining to detect intracellular ROS levels. As shown in Figure 4K, LPS treatment significantly increased DHE fluorescence intensity, indicating enhanced ROS production (*p* < 0.01 vs. sham and LPS + SP16 group; *p* < 0.001 vs. SP16 group) (Figure 4L). This increase was notably attenuated by SP16 treatment, suggesting its protective role against oxidative stress.

### 3.5 Targeted Metabolomics Study Suggested SP16 May Modulate Glucose Metabolism

Metabolomic profiling provided insights into the physiological impact of SP16. A total of 67 metabolites were identified, and their variations were assessed using multivariate statistics (Table S1). While PCA indicated global differences, PLS‐DA and OPLS‐DA models maximized group separation, demonstrating distinct clustering between the LPS and LPS + SP16 cohorts (Figure S2A–C). The robustness of these models was confirmed by permutation tests, where high R2Y and Q2 values established the reliability of the observed metabolic distinctions (Figure S3).

Heatmap analysis visualized the reversal of specific metabolic alterations by SP16 (Figure S2D). We identified 15 significant differential metabolites (*p <* 0.05, VIP > 1.0) (Table S2), with fructose 1,6‐bisphosphate (FBP) showing the most prominent upregulation in the SP16‐treated group (Figure S2E). KEGG pathway enrichment corroborated these observations, showing that differential metabolites were mapped principally to the glucagon signaling pathway, TCA cycle, and oxidative phosphorylation (Figure S2F). Given that FBP is pivotal for glycolytic flux and oxidative balance, its restoration suggests a potential improvement in immune‐metabolic function. We performed an ELISA to validate the level of FBP. In the LPS group, FBP increased higher than sham and SP16 (both *p* < 0.05). While LPS + SP16 also increased FBP compared with sham and SP16 (both *p* < 0.001) (Figure S4). Further analysis of glucose metabolism sub‐pathways (Figure S5A–F) revealed obvious changes in the abundance of Pentose Phosphate Pathway (PPP) products. These results highlight the capacity of SP16 to modulate glucose utilization and improve central energy regulation.

### 3.6 Transcriptomics Study Revealed the Potential Role of SP16 in Immune‐Modulating Effect

**FIGURE 5:**
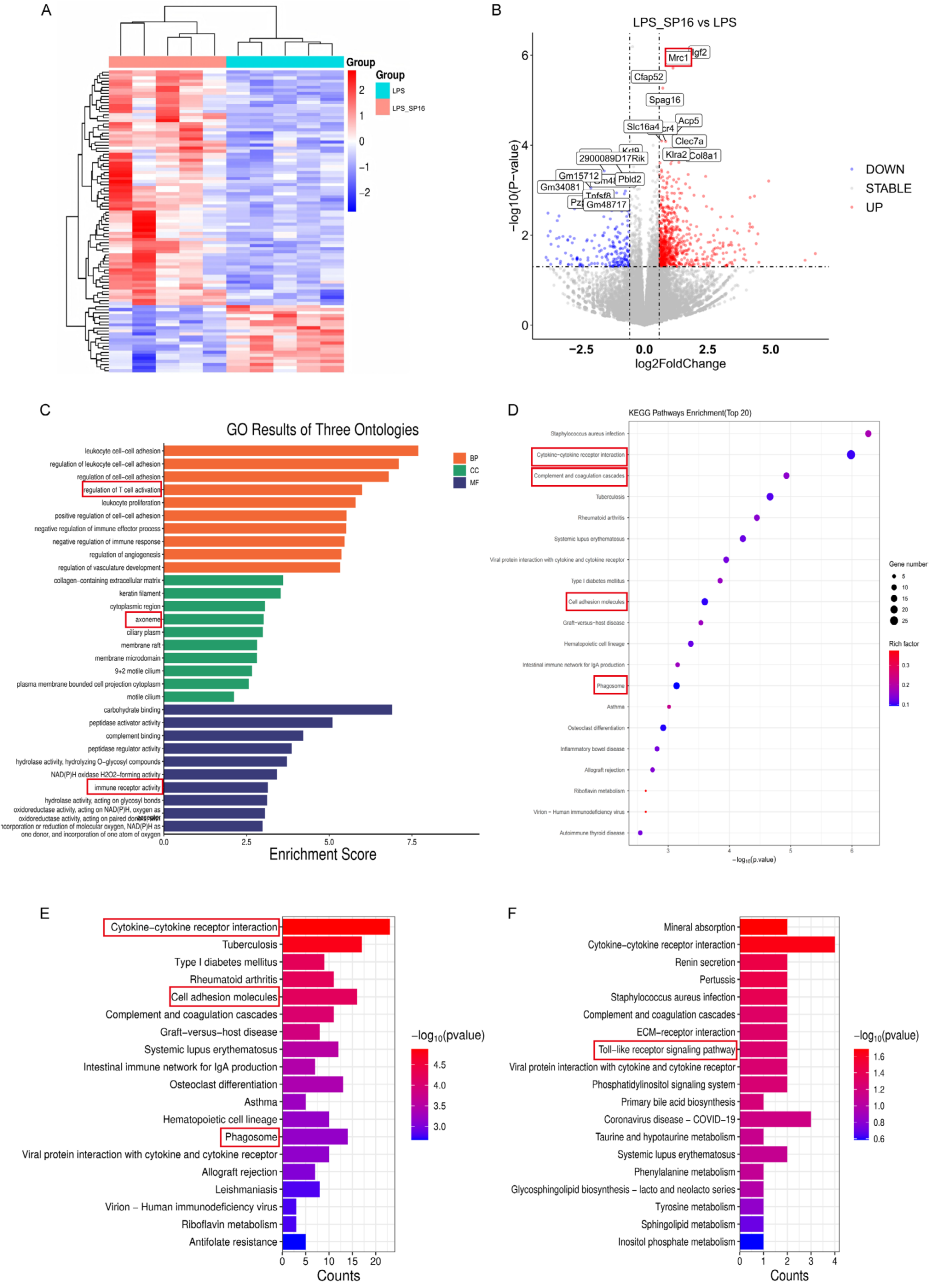
Transcriptomic profiling uncovers the immunomodulatory mechanism of SP16. (A) Hierarchical clustering heatmap showing the expression patterns of differentially expressed genes (DEGs) between groups. (B) Volcano plot displaying the distribution of significant DEGs; red dots indicate upregulation and blue dots indicate downregulation. (C) Gene Ontology (GO) functional enrichment analysis of the DEGs. (D) KEGG pathway enrichment analysis for all significant genes. (E, F) Directed KEGG pathway analysis specifically for (E) upregulated and (F) downregulated genes.

To map the transcriptomic landscape remodeled by SP16, we performed RNA‐Seq on hippocampal tissues. Following rigorous quality control (46–59 million clean reads/sample; Table S3), the dataset revealed distinct expression profiles between groups. Hierarchical clustering highlighted a subset of genes dysregulated by LPS but effectively restored by SP16 intervention. In total, 940 differentially expressed genes (DEGs) were identified, comprising 741 upregulated and 199 downregulated transcripts (Figure 5A). Notably, SP16 treatment upregulated key genes associated with immune modulation and tissue regeneration, such as *Mrc1*, *Igf2*, *Slc16a4*, and *Clec7a* (Figure 5B), suggesting a shift toward a reparative microenvironment.

Pathway enrichment analyses (GO and KEGG) provided a mechanistic context for these changes. While LPS alone triggered widespread inflammatory disruption, SP16 treatment significantly modulated pathways related to “complement and coagulation cascades” and “phagosome” function (Figure 5C,D). Crucially, SP16 administration dampened the pro‐inflammatory “toll‐like receptor signaling pathway” while simultaneously enhancing cell adhesion and phagocytic processes (Figure 5E,F). These patterns indicate that SP16 mitigates LPS‐induced neuroinflammation by reprogramming microglial responses, specifically promoting a reparative signature, likely via the LRP1 signaling axis.

### 3.7 Multi‐Omics Integration of Transcriptomic and Metabolomic Data

**FIGURE 6:**
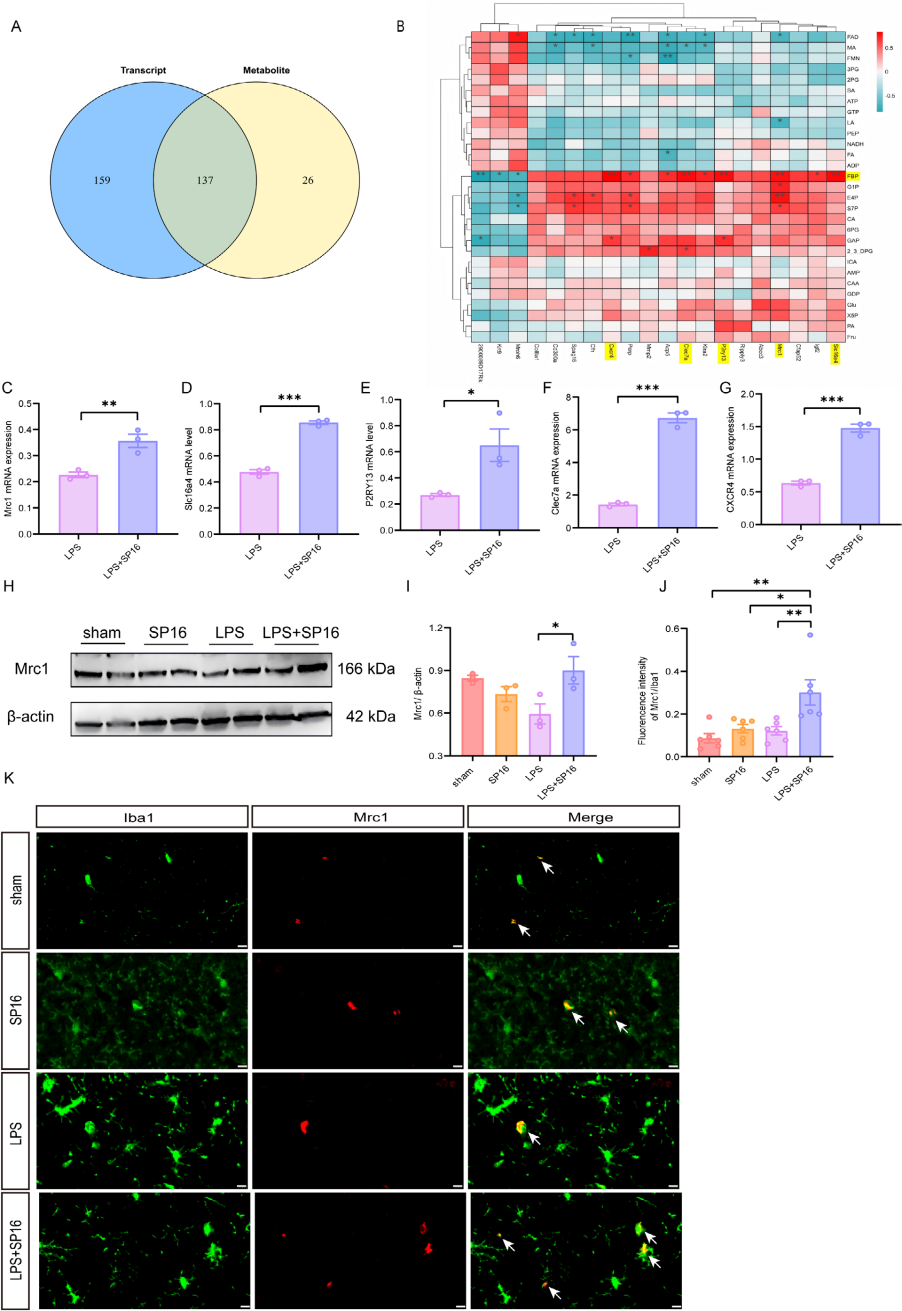
Multi‐omics integration of transcriptomic and metabolomic data. (A) Venn diagram illustrating the intersection of significantly enriched pathways (or features) between the transcriptomic and metabolomic datasets. (B) Correlation heatmap matrix displaying the association between differentially expressed genes (DEGs) and differential metabolites. Red indicates positive correlation, while blue indicates negative correlation. (C–G) Relative mRNA expression levels of selected DEGs (*Mrc1, Slc16a4, P2ry13, Clec7a*, and *Cxcr4*) were determined by RT‐qPCR. (H, I) Representative Western blots and quantitative analysis of Mrc1 expression (*n* = 3). (J) Quantitative analysis of fluorescence intensity of Mrc1 expression within the microglial population. (K) Representative double‐immunofluorescence staining for Mrc1 (target) and Iba1 (microglia). Scale bars = 10 μm. Data are presented as mean ± SEM. *p < 0.05, **p < 0.01, ***p < 0.001.

To decode the systemic interplay between gene regulation and metabolic flux, we integrated transcriptomic and metabolomic datasets onto shared KEGG pathways. This multi‐omics approach identified 137 overlapping pathways functioning at both the transcriptional and metabolic levels (Figure 6A). By mapping significantly altered candidates (*p <* 0.05) into a correlation matrix, we generated a heatmap (Figure 6B) that visualizes the robust network connections between differential metabolites and genes. Crucially, the analysis suggests potential positive correlations between SP16‐regulated genes and key intermediates, specifically FBP, erythrose 4‐phosphate (E4P), and sedoheptulose 7‐phosphate (S7P). These multi‐dimensional signatures suggest that SP16 confers neuroprotection by restoring glucose homeostasis and reversing LPS‐induced metabolic dysregulation. Specifically, the tight association between FBP and genes such as *Mrc1*, *Slc16a4*, *P2ry13*, *Clec7a*, and *Cxcr4* highlights how transcriptional changes modulate metabolic output.

To validate these differentiated genes, we confirmed candidate gene expression using RT‐qPCR. Consistent with the sequencing data, SP16 significantly upregulated the mRNA levels of immune‐modulators, including *Mrc1*, *Slc16a4*, *P2ry13*, *Clec7a*, and *Cxcr4* compared to the LPS group (*p <* 0.05; Figure 6C–G).

We subsequently verified Mrc1 upon LRP1 activation at the protein level. As illustrated in Figure 6H,I, the LPS + SP16 group exhibited a marked upregulation of Mrc1 compared to the LPS cohort (*p* < 0.05). These quantitative findings were closely corroborated by immunofluorescence staining, which demonstrated that SP16 modulates immune responses during neuroinflammation (Figure 6J,K). LPS stimulation induced a moderate compensatory increase in the expression of Mrc1 (*p* < 0.01 vs. sham and SP16). Crucially, SP16 intervention further synergized with this response, severely amplifying Mrc1 expression. The LPS + SP16 group displayed an expanded Mrc1‐positive cell population and significantly higher Mrc1 fluorescence intensity than sham (*p* < 0.01), SP16 (*p* < 0.05), and the LPS group (*p* < 0.01).

## 4 Discussion

The present study elucidates the novel mechanistic insights into brain insulin resistance and cognitive dysfunction following neuroinflammation. Systemic LRP1 activation via SP16 confers neuroprotection, attenuating neuroinflammation and cognitive impairment. Integrating metabolomic and transcriptomic datasets, we traced this protective effect to the modulation of glucose metabolism and the promotion of Mrc1 expression. These observations prompted us to further interrogate the immunomodulatory role of LRP1.

LRP1 has emerged as a pleiotropic hub governing neuroprotection, yet its pharmacological targeting in acute neuroinflammation remains underutilized. This study provides novel evidence that SP16, a serpin‐derived LRP1 agonist, effectively rescues cognitive phenotypes in an LPS‐induced model, bridging the gap between systemic immunomodulation and central neuroprotection. While previous investigations established SP16's efficacy in mitigating cardiac ischemia–reperfusion injury [27] and generic neurological deficits [24, 30], we extend its therapeutic narrative to the highly vulnerable hippocampal circuitry.

Mechanistically, a critical finding is SP16's capacity to interrupt the glial inflammatory axis. LPS typically stimulates microglia to secrete pro‐inflammatory cytokines (TNF‐α, IL‐1β, and IL‐6), which subsequently activate astrocytes [18]. Through comprehensive multi‐modal analyses, we demonstrated that SP16 firmly attenuates the activation of both microglia and astrocytes in the hippocampus, thereby reversing this destructive cytokine surge. Crucially, these molecular improvements directly translate into enhanced neuronal survival and cognitive recovery. Notably, we observed a behavioral dissociation: While SP16 significantly restored spatial memory retention (evidenced by rescued platform crossings), overall swimming speed in the MWM and total arm entries in the Y‐maze remained diminished. This divergence suggests that at the 24‐h peak phase, SP16 mitigates neurocognitive impairment, even while acute, systemic sickness behavior persists. The recovery of platform crossings strongly substantiates SP16's specific neuroprotective efficacy against cognitive insults. Nevertheless, our complete behavioral and molecular findings firmly establish SP16‐mediated LRP1 activation as a potent strategy to quell the neuroinflammatory cytokine surge and preserve cognitive function.

To define the molecular basis of SP16‐linked neuroprotection, we examined the insulin signaling cascade, a key regulator of neuronal survival and synaptic plasticity. The PI3K/AKT pathway is traditionally linked to metabolic homeostasis, but in the central nervous system, it also inhibits apoptosis and promotes neurite outgrowth [31, 32, 33]. Our data indicate that SP16‐induced LRP1 activation restores this pathway in the inflamed hippocampus. LPS challenge markedly suppressed AKT phosphorylation, a deficit that SP16 treatment effectively promoted. This finding aligns with Stefano et al., who showed that SP16 confers cardio‐protection via AKT signaling in acute myocardial infarction models [27]. Therefore, our data suggest that SP16 does not merely act as a general anti‐inflammatory agent but modulates neuroprotection and rescues spatial recognition memory by normalizing the LRP1‐associated central insulin signaling network.

We further identified GSK‐3β as a critical downstream effector. Pathological overactivation of GSK‐3β, resulting from reduced inhibitory phosphorylation at Ser9, drives Tau hyperphosphorylation and mitochondrial dysfunction [34]. SP16 restored the inhibitory phosphorylation of GSK‐3β, thereby limiting its activity. Importantly, our findings demonstrate that the LRP1‐AKT axis is conserved in the CNS, indicating that SP16's cytoprotective effects are pathway‐specific rather than tissue‐specific. This broadens its potential as a therapeutic strategy for ischemic or inflammatory conditions.

The metabolic dysfunction induced by LPS stems largely from mitochondrial impairment, which not only limits energy production but also drives neuronal toxicity [35]. Loss of mitochondrial integrity leads to ROS accumulation that exceeds cellular antioxidant capacity, resulting in oxidative stress [36]. Our biochemical data reflect this process: LPS caused a sharp decline in SOD and GSH levels while elevating hydrogen peroxide. SP16 treatment attenuated these changes and functionally restored redox homeostasis. Meanwhile, the results of T‐AOC revealed that SP16 promoted the tissue's antioxidant capacity. This finding has important clinical implications. Direct antioxidant therapies, such as Vitamin E or polyphenols, have largely failed in neuroprotection trials due to poor CNS penetration or stoichiometric limitations [37, 38]. In contrast, SP16 engages LRP1 to activate a sustained, endogenous defense program, offering greater biological durability than exogenous radical scavengers.

The restoration of redox balance also provides a mechanistic link to the improved insulin signaling reported in our previous work [17]. Oxidative stress inhibits metabolic signaling: Excessive ROS activates stress kinases such as JNK, which uncouple insulin receptor function [39]. By normalizing the oxidative environment, SP16 relieves this inhibition and resensitizes the PI3K/AKT pathway. Thus, SP16 does not address isolated symptoms but rather disrupts the pathological cycle in which inflammation promotes oxidative stress, which in turn exacerbates neuronal insulin resistance.

The intersection of neuroinflammation and central insulin resistance fundamentally alters how brain cells handle glucose. To dissect this metabolic reprogramming and evaluate SP16's restorative potential, we performed targeted metabolomics. Rather than a global metabolite shift, changes were highly concentrated among glycolytic intermediates, with fructose‐1,6‐bisphosphate (FBP) emerging as the most responsive target. From an immune‐metabolic perspective, FBP is recognized not simply as a fuel substrate but as an active signaling hub.

Interestingly, our validation revealed an intriguing dynamic: While LPS treatment elevated FBP levels, reflecting a shift toward aerobic glycolysis to meet acute inflammatory energy demands, the LPS + SP16 treatment resulted in an even greater accumulation of FBP. This remarkable accumulation following SP16 intervention likely reflects an LRP1‐induced, targeted metabolic defense mechanism rather than a pathological buildup. High FBP levels engage a feed‐forward loop through PFK1 and the PI3K/AKT axis, actively locking cells into a survival state [40]. Since FBP possesses intrinsic neuroprotective, anti‐inflammatory, and antioxidant properties, its deliberate accumulation serves a critical purpose. It acts as a transient metabolic bottleneck that strategically shunts upstream glucose flux into the pentose phosphate pathway. This rerouting is crucial for generating NADPH to combat LPS‐induced ROS, which aligns with the attenuated oxidative stress observed in our SP16‐treated mice.

Importantly, synchronous changes in TCA cycle metabolites (such as citrate and α‐ketoglutarate) indicate that this upstream rerouting does not stall downstream energy production; rather, SP16 enhances overall metabolic flux. Since FBP dynamics correlate tightly with synaptic protein synthesis and its dysregulation is linked to Alzheimer's pathology [41], modulating this crucial node allows SP16 to relieve the metabolic inflexibility that contributes to cognitive decline.

Broadening the scope, these specific fluctuations in FBP were echoed by systemic shifts in our multivariate analysis. The separation between groups in the PCA plots reflects a stress‐dependent metabolic signature. This interpretation is reinforced by our KEGG enrichment data, which confirmed that the therapeutic impact of SP16 is centered on the restoration of core glucose utilization pathways, specifically the citrate cycle, pentose phosphate pathway, and oxidative phosphorylation. Thus, SP16 appears to rescue the brain from metabolic exhaustion, re‐establishing the efficient energy cycles necessary for neuronal survival.

Microglial plasticity is central to the cognitive deficits observed in neuroinflammatory conditions, including PND [42]. Far from being static responders to injury, these immune sentinels operate across a dynamic activation spectrum [43]. The pathological bottleneck arises when microglia become entrenched in a pro‐inflammatory (M1‐like) phenotype, perpetuating neurotoxicity rather than supporting tissue health. Consequently, therapeutic success depends not merely on suppression, but on actively driving repolarization toward a restorative (M2‐like) state [44].

Our transcriptomic data suggest a potential link between SP16‐mediated LRP1 activation and the modulation of this effect. Specifically, volcano plots reveal a distinct upregulation of *Mrc1*, a canonical signature of the anti‐inflammatory phenotype [45]. This indicates that LRP1 signaling goes beyond dampening inflammation; it fundamentally reprograms the hippocampal transcriptional landscape to favor resolution. Pathway analyses further elucidate a dual mechanism: KEGG enrichment confirms the downregulation of toll‐like receptor (TLR) signaling, effectively severing the LPS toxicity loop, while GO analysis highlights a simultaneous upregulation of repair‐oriented pathways, including phagosome activity and axoneme regulation. Thus, SP16 mediates a coordinated biological pivot: Silencing acute innate immunity while enabling the debris clearance necessary for neurovascular restoration.

This broad immunomodulatory effect is further corroborated by the differential expression of key genes implicated in other neuropathology situations. For instance, the modulation of chemokines and ligands parallels mechanisms seen in multiple sclerosis and Alzheimer's disease [46]. We observed expression patterns in *Cxcr4* and *Slc16a4* consistent with immune cell infiltration regulation [47, 48], alongside changes in *P2RY13* and *Clec7a*, biomarkers typically associated with the aberrant microglial activation found in sepsis‐associated encephalopathy [49, 50]. These findings collectively underscore the comprehensive nature of LRP1‐associated immune microenvironment remodeling.

The multi‐omics approach provides a comprehensive understanding of the molecular mechanisms involved, highlighting SP16's ability to restore neuronal function and metabolic balance. Based on these findings, future efforts could be focused on the exploration of the specific molecular interactions between genes and metabolites modulated by SP16, the investigation of the long‐term efficacy and safety of SP16 in improving cognitive function, and the extension of these findings to human models to evaluate LRP1's potential as a therapeutic intervention for neuroinflammation‐associated complications.

Despite its comprehensive insights, our study has four main limitations. First, using whole hippocampal tissue lacks cell‐type‐specific resolution. Given LRP1's broad expression, untangling the unique contributions of microglia vs. neurons and astrocytes will require future single‐cell RNA sequencing or flow‐sorted isolation. Second, our mechanistic findings remain largely associative. Metabolic targets like FBP were identified via multi‐omics correlations. Without gain‐ or loss‐of‐function validation, future experiments are imperative to confirm whether these metabolic shifts directly mediate SP16's therapeutic effects. Third, the pharmacological administration route introduces ambiguity. Systemic SP16 delivery, coupled with a lack of microglial‐specific LRP1 knockouts, makes it impossible to distinguish direct central nervous system engagement from peripheral anti‐inflammatory effects. Future studies must employ labeled tracers and conditional knockouts to clarify these spatiotemporal dynamics. Lastly, our experimental model restrict generalizability. The acute, self‐limiting LPS model precludes evaluating long‐term functional recovery, while the exclusive use of male mice overlooks well‐documented sexual dimorphism in neuroinflammation. Future research advancing clinical translation must prioritize chronic neurodegenerative models, inclusive sex cohorts, and cell‐specific targeting strategies.

## 5 Conclusion

In conclusion, LRP1 is downregulated during acute neuroinflammation. Pharmacological activation of LRP1 with SP16 protects against insulin resistance and cognitive impairment. Mechanistically, SP16 mitigates LPS‐induced neuroinflammation by activating PI3K/AKT/GSK‐3β signaling, reducing oxidative stress, and increasing FBP generation. Furthermore, it promotes the microglial transcriptomic profile toward a reparative state, evidenced by upregulated Mrc1 (Figure S6). Ultimately, these synergistic effects position the LRP1 agonist SP16 as a compelling therapeutic candidate for further investigation in cognitive management.

## Author Contributions

W.M. and Y.M. conceived and designed the study. M.Q., Y.H., and L.Y. drafted the manuscript. Y.L. and H.Y. revised the manuscript. R.W., Y.L., and M.S. analyzed the data. W.M. was the guarantor of the study and had full access to all the study data. All the authors have read and approved the submitted version.

## Funding

This work was supported by the National Natural Science Foundation of China (Nos. 82171180 and 82371469).

## Ethics Statement

Mice were obtained from the Laboratory Animal Center of the China PLA General Hospital. All experimental procedures were approved by the Animal Ethics Committee of the Chinese PLA General Hospital (2021‐X16‐88).

## Conflicts of Interest

The authors declare no conflicts of interest.

## Supporting information


**Figure S1:** Additional behavior test data and TUNEL staining of the hippocampus.
**Figure S2:** Targeted metabolomics reveals that SP16 modulates glucose metabolism.
**Figure S3:** Permutation test validation of the OPLS‐DA model.
**Figure S4:** Validation of the level of fructose‐1,6‐bisphosphate.
**Figure S5:** Alterations in glucose metabolism profiles between the LPS and LPS_SP16 groups.
**Figure S6:** Schematic illustration of the neuroprotective mechanisms of SP16 against LPS‐induced cognitive impairment.

**Table S1:** Annotated quantity results of metabolites.

**Table S2:** Annotated differently expressed metabolites with VIP.

**Table S3:** Read QC (clean reads).

## Data Availability

The data that support the findings of this study are available on request from the corresponding author. The data are not publicly available due to privacy or ethical restrictions.
