# Supplementary material for: LRP1 Activation Promotes Metabolic Reprogramming and Mrc1 Expression to Attenuate LPS‐Induced Cognitive Deficits: An Integrated Omics Analysis
Source: CNS Neurosci Ther. 2026 May 29;32(6):e70950. doi: 10.1002/cns.70950 (PMC13239108; doi:10.1002/cns.70950)
Supplement: Supplementary file 1 — Figure S1: Additional behavior test data and TUNEL staining of the hippocampus. Figure S2: Targeted metabolomics reveals that SP16 modulates glucose metabolism. Figure S3: Permutation test validation of the OPLS‐DA model. Figure S4: Validation of the level of fructose‐1,6‐bisphosphate. Figure S5: Alterations in glucose metabolism profiles between the LPS and LPS_SP16 groups. Figure S6: Schematic illustration of the neuroprotective mechanisms of SP16 against LPS‐induced cognitive impairment. [file CNS-32-e70950-s002.docx]

**Supplementary materials**

**Figure S1. Additional behavior test data and TUNEL staining of the hippocampus.
A.** Average swimming speed (m/s) of mice in the probe test of MWM.

1. Platform-site crossings during the probe test.
2. Total entries of old and new arms in the Y-maze.
3. TdT-mediated dUTP nick end labeling (TUNEL) staining of the hippocampus to visualize neural apoptosis in the different groups.
4. Statistical analysis of TUNEL positive areas among the groups.

**
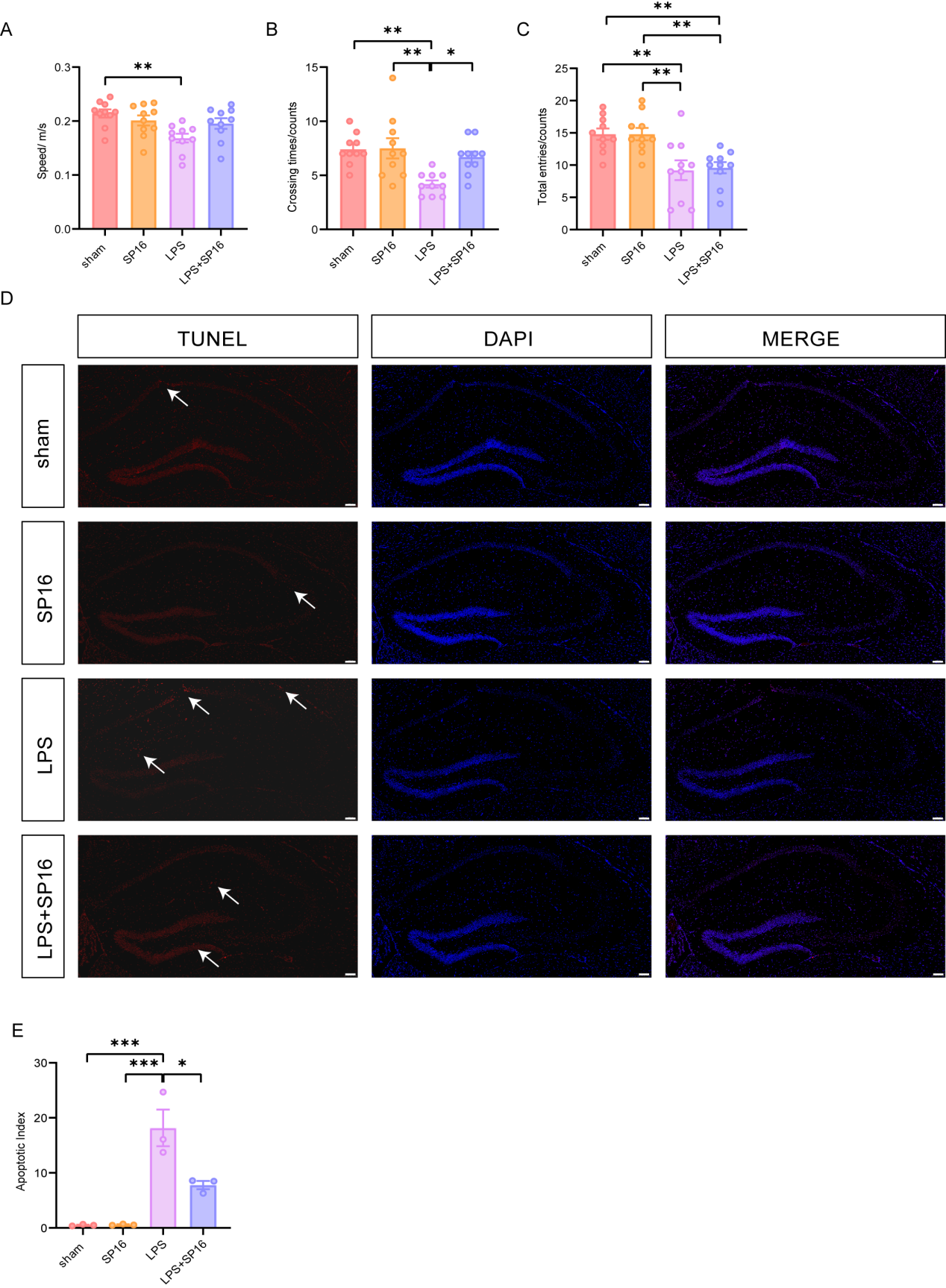
**

**Figure S2. Targeted metabolomics reveals that SP16 modulates glucose metabolism.****A** PCA score plot displaying the overall clustering of metabolic profiles across different groups.
**B** PLS-DA score plot illustrating biochemical distinction and group separation.
**C** OPLS-DA score plot highlighting the maximization of inter-group differences.
**D** Hierarchical clustering heatmap of differential metabolites between the LPS and LPS+SP16 groups.
**E** Visualization of significantly altered metabolites (LPS vs. LPS+SP16). Red indicates upregulation, and green indicates downregulation.
**F** Bubble plot of KEGG pathway enrichment analysis. Bubble size represents the number of enriched metabolites, and the color gradient depicts statistical significance (p-value)


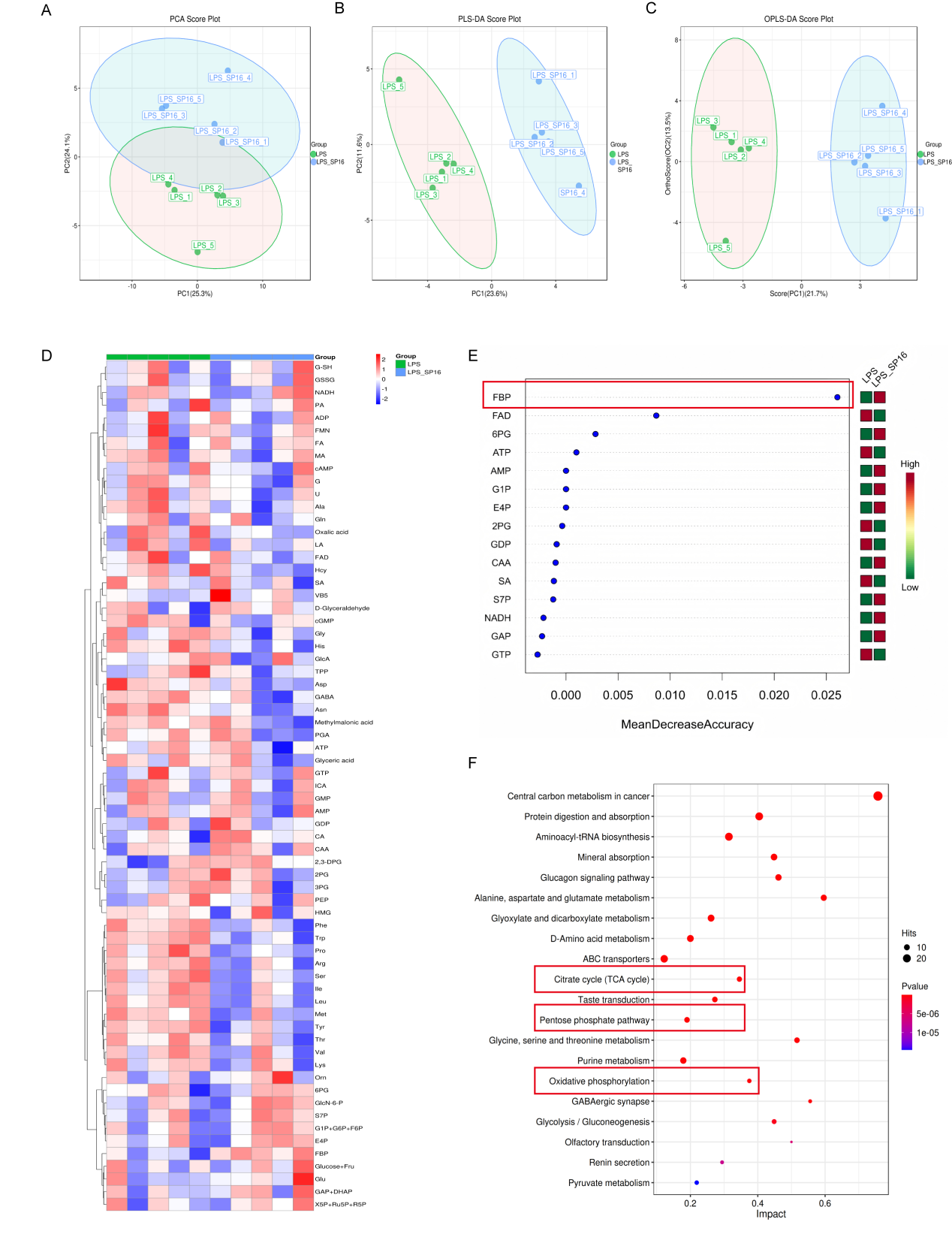


**Figure S3. **Permutation test validation of the OPLS-DA model.****
The model’s reliability was validated using a permutation test with 200 iterations (the number of permutations should match your actual settings). The horizontal axis represents the correlation between the permuted class labels and the original labels. The vertical axis indicates the R^2^ (green circles) and Q^2^ (blue squares) values. The two dashed lines represent the linear regression of R^2^ and Q^2^. As shown in the plot, all R^2^ and Q^2^ values on the left are lower than the original points on the right. The intercepts of the R^2^ and Q^2^ regression lines on the y-axis are 0.9 and -0.09, respectively. The results (Q^2^ intercept < 0) demonstrate that the OPLS-DA model is robust and lacks overfitting, indicating the high predictive capability of the model.

**
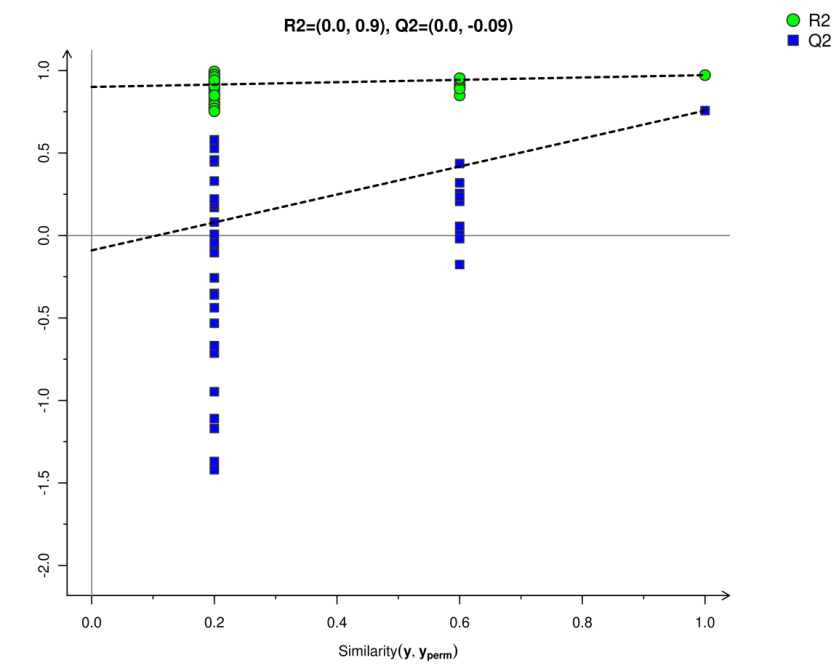
**

**Figure S4. Validation of the level of **fructose-1,6-bisphosphate.****

**
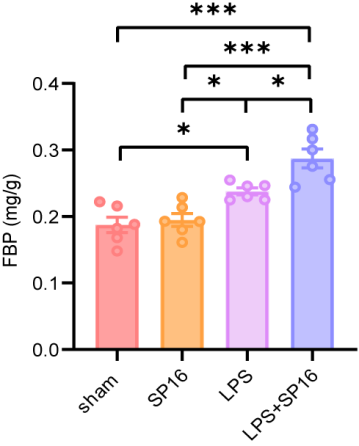
**

**Figure S5. **Alterations in glucose metabolism profiles between the LPS and LPS_SP16 groups.****
****A**** The relative abundance of the identified key metabolites in individual samples from the LPS and LPS_SP16 groups (n=5 per group). The stacked bar chart illustrates the compositional proportion of 20 categorized metabolites, with each color corresponding to a specific metabolite as indicated in the legend.
****B**** Schematic overview of the centralized metabolic network, encompassing glycolysis, the pentose phosphate pathway, and the TCA cycle. The inset bar graphs adjacent to the nodes represent the relative levels of the corresponding intermediate metabolites between the two groups.
****C–F**** Hierarchical clustering heatmaps showing the relative abundance of metabolites involved in Glycolysis, the Pentose phosphate pathway, the TCA cycle, and energy metabolism. Each column represents an individual biological replicate (cyan indicating the LPS group and pink indicating the LPS_SP16 group), and each row represents a distinct metabolite. The color scale reflects the strictly normalized expression values, where red indicates higher abundance and blue indicates lower abundance.

**
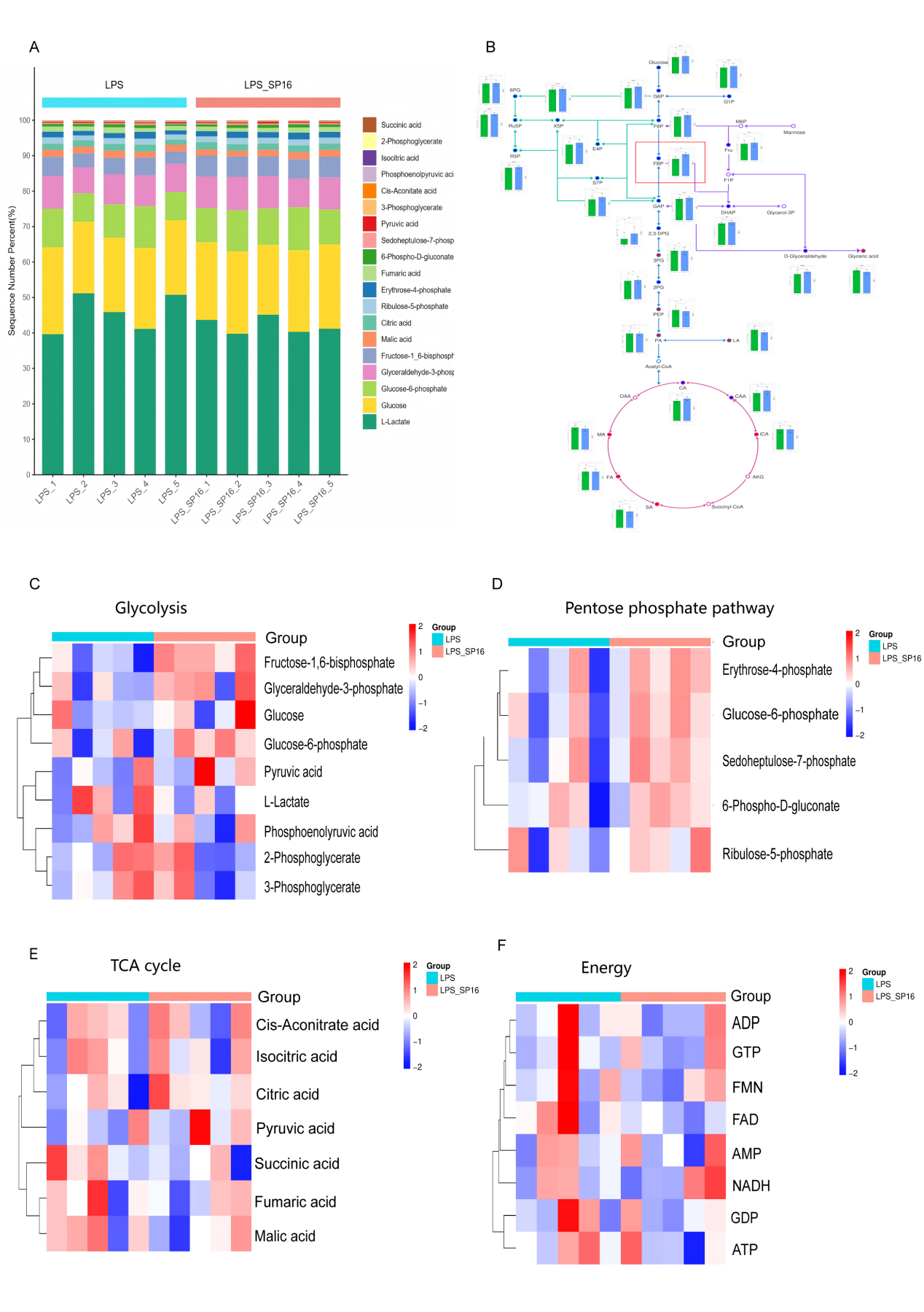
**

**Figure S6. Schematic illustration of the neuroprotective mechanisms of SP16 against LPS-induced cognitive impairment.**

Under pathological conditions, LPS initiates a cascade involving the infiltration of damaging peripheral immune cells and the release of inflammatory mediators, which triggers microglial overactivation in the hippocampus and ultimately leads to cognitive dysfunction. Pharmacological activation of LRP1 by SP16 counteracts this process. SP16 orchestrates neuroprotection by modulating the PI3K/AKT/GSK-3β signaling axis, reducing oxidative stress, and enhancing FBP generation. Crucially, these molecular changes drive a phenotypic shift toward M2-like microglia, thereby resolving hippocampal inflammation and alleviating insulin resistance. Collectively, these findings position the LRP1 agonist SP16 as a compelling therapeutic candidate for cognitive management.


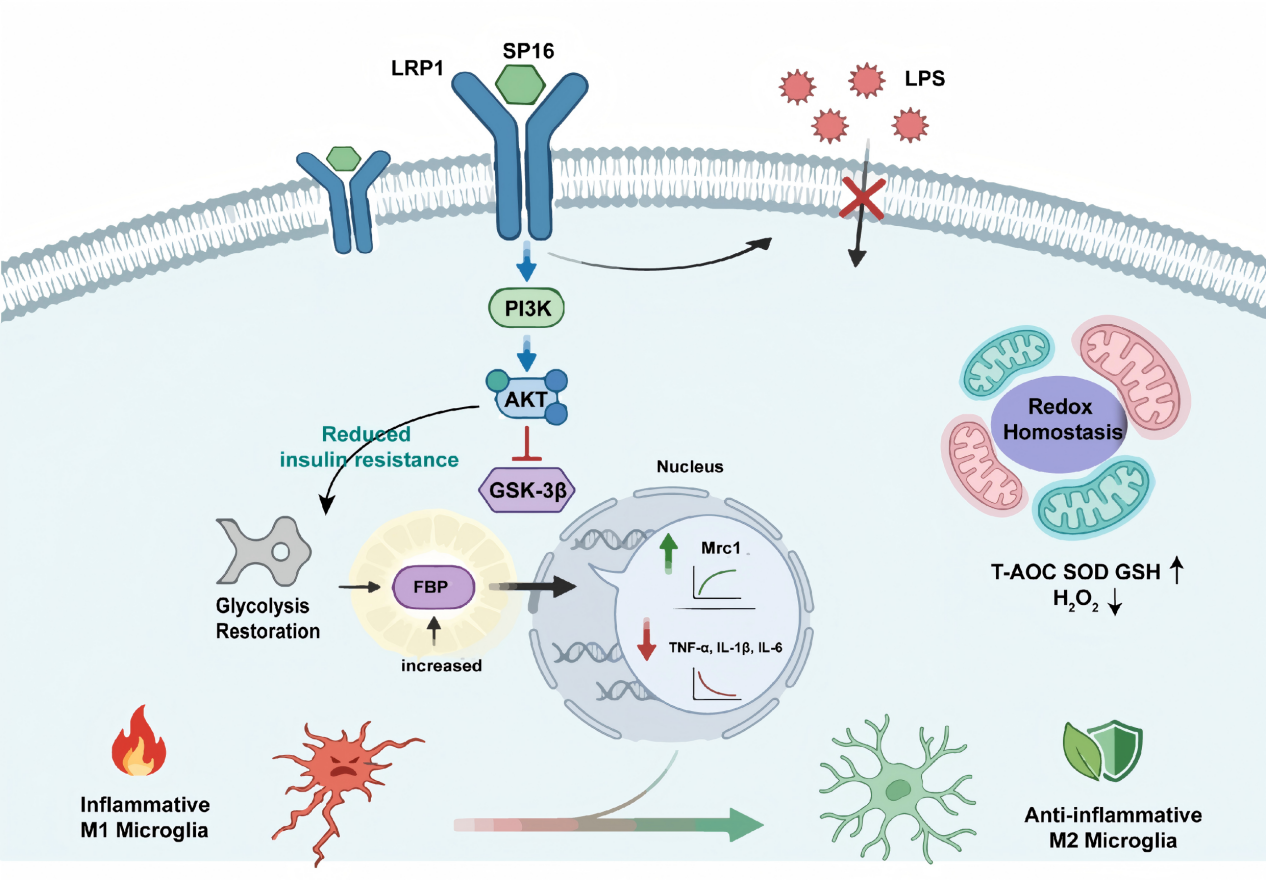


**Table S1 annotated quantity results of metabolites.**

xls file in Supplementary materials.

**Table S2 annotated differently expressed metabolites with VIP.**

xls file in Supplementary materials.

**Table S3 Read QC (clean reads).**

xls file in Supplementary materials.
